# CT-Angiographie zum Nachweis des zerebralen Zirkulationsstillstandes: Aktualisierung der Kriterien

**DOI:** 10.1007/s00062-022-01229-z

**Published:** 2022-11-15

**Authors:** Heinrich Lanfermann, Stephan A. Brandt

**Affiliations:** 1 Institut für Diagnostische und Interventionelle Neuroradiologie, Medizinische Hochschule Hannover, Carl-Neuberg-Str. 1, 30625 Hannover, Deutschland; 2 Klinik für Neurologie, Charité Campus Mitte, Charitéplatz 1/Bonhoefferweg 3, 10117 Berlin, Deutschland

Die CT-Angiographie (CTA) ist seit 2015 [1] als ergänzendes Verfahren zur Feststellung des zerebralen Zirkulationsstillstandes in Deutschland zugelassen. Die Vorgaben zu Durchführung und Auswertung der CTA [2, 3] wurden entsprechend den Empfehlungen eines Cochrane-Reviews [4] dabei umfassend festgelegt. Dies war erforderlich, um zu vermeiden, dass ein zerebraler Zirkulationsstillstand und damit ein irreversibler Hirnfunktionsausfall mittels CTA diagnostiziert wird, obwohl dieser nicht vorliegt. Diese zwingende Voraussetzung für die Einführung der CTA im Rahmen der Feststellung des zerebralen Zirkulationsstillstandes resp. Hirnfunktionsausfalles wurde zuverlässig eingehalten. Es sind keine Fälle bekannt geworden, bei denen trotz korrekter Anwendung ein falsch-positiver Befund erhoben worden wäre. Damit hat sich die CTA für diese Fragestellung im klinischen Einsatz bewährt. Sie ist zu einem wichtigen und inzwischen häufig angewandten apparativen ergänzenden Untersuchungsverfahren geworden, mit dem die Wartezeit bei Nachweis des zerebralen Zirkulationsstillstandes auch unter erschwerten Bedingungen verkürzt werden kann.

Die Bundesärztekammer hat gemäß dem Transplantationsgesetz den gesetzlichen Auftrag, den Stand der Erkenntnisse der medizinischen Wissenschaft regelmäßig zu überprüfen, und hat aktuell die „Richtlinie zur Feststellung des irreversiblen Hirnfunktionsausfalls“ – früher als Hirntoddiagnostik bezeichnet – fortgeschrieben [5].

## Aktuelle Neuerungen

Insbesondere während der COVID-19-Pandemie mussten zahlreiche PatientInnen mittels extrakorporaler Membranoxygenierung (ECMO) behandelt werden. Abhängig vom Ausmaß der Erkrankung wird entweder ein Lungenbypass, die sog. venovenöse ECMO (vv-ECMO), oder ein Herz- und Lungenbypass, die sog. venoarterielle ECMO (va-ECMO), angelegt. Abhängig vom Ort der jeweiligen Kanülierung könnte es bei der va-ECMO zu einer asymmetrischen oder sogar einseitigen Kontrastmittelanflutung der Kopf- und Halsgefäße kommen [6]. Zudem entspricht der individuell variierende zeitliche Ablauf der Kontrastmittelanflutung bei PatientInnen mit einer va-ECMO nicht immer dem bei PatientInnen mit erhaltener Herz- und Lungenfunktion. Da für die in Deutschland geltenden CTA-Kriterien nur die arterielle Phase der CTA, die eine zeitgerechte Kontrastmittelanflutung erfordert, gewertet wird,* ist bei PatientInnen mit gleichzeitiger va-ECMO eine zuverlässige Beurteilung der CTA zur Feststellung des zerebralen Zirkulationsstillstandes nicht möglich *[5*S. 9 und 14*]*.*

Bei PatientInnen mit einer vv-ECMO (nur Lungenbypass) besteht diese Einschränkung nicht. Entsprechend *kann die CTA zur Feststellung des zerebralen Zirkulationsstillstandes bei PatientInnen mit einer vv-ECMO eingesetzt werden. Voraussetzung ist jedoch, dass ein Spezialist für ECMO-Verfahren die CTA-Untersuchung begleitet und nach Rücksprache mit den verantwortlichen (Neuro‑)Radiologen und MTRA die korrekte Kontrastmittelgabe sicherstellt.*

## Ergänzung der Empfehlung bei bestehenden Kalottendefekten

Traumatisch oder operativ bedingte Defekte der Schädelkalotte können den Anstieg des intrakraniellen Druckes z. B. durch ein Hirnödem oder eine Blutung verzögern und ggf. verhindern, dass der intrakranielle Druck über den arteriellen Druck steigt. Entsprechend tritt unter diesen Bedingungen kein zerebraler Zirkulationsstillstand ein. In einer Studie zur Sensitivität der CTA zum Nachweis des zerebralen Zirkulationsstillstandes bei Patienten mit Kraniotomien oder Kraniektomien konnten Nunes et al. [7] zeigen, dass mittels CTA ein zerebraler Zirkulationsstillstand nachweisbar ist, allerdings die Sensitivität in Abhängigkeit von der Größe des Kalottendefektes geringer ist. Im Vergleich zu Patienten mit intakter Schädelkalotte lag bei Patienten mit einer Kraniotomie die Sensitivität bei 87,5 % und bei Patienten mit einer Kraniektomie bei 60 %. Entsprechend kann die CTA auch bei Patienten mit Kalottendefekten eingesetzt und ein Zirkulationsstillstand nachgewiesen werden. Allerdings ist die Sensitivität insbesondere aufgrund von verstärkt auftretendem „stasis filling“ nach Angaben der Autoren relativ reduziert.

## Noch erforderliche Bestätigungen

Das bereits aus der konventionellen Angiographie bekannte Stasis filling, eine langsam progrediente Ausbreitung des Kontrastmittels in den intrakraniellen Arterien bei sistierender zerebraler Zirkulation, wurde innerhalb des Arbeitskreises zur Aktualisierung der Richtlinien [5] zur Feststellung des irreversiblen Hirnfunktionsausfalles erneut sorgfältig diskutiert. Suarez-Kelly et al. [8] hatten als Studienergebnis mitgeteilt, dass ein intraarterieller Schwellenwert von 80 Hounsfield Units (HU) für die Unterscheidung von erhaltener Hirndurchblutung und sistierender zerebraler Zirkulation (Stasis filling) geeignet sei. Ein in dieser Größenordnung gelegener Schwellenwert war auch von Welschehold et al. [9] festgestellt worden. Die aktuelle Studienlage wurde innerhalb des Arbeitskreises allerdings als noch nicht ausreichend gewertet, um einen exakten Grenzwert in Hounsfield Units für den Nachweis eines Stasis filling in der CTA festzulegen.

Sorgfältig geprüft wurde zudem das Ergebnis einer Studie von Kerhuel et al. [10], in der eine Zunahme der Sensitivität der CTA in Abhängigkeit vom Zeitpunkt der Durchführung der CTA berichtet wurde. Die Datenlage wurde als nicht ausreichend gewertet, um ein definitives Zeitfenster für die Durchführung der CTA festzulegen.

Die Kriterien für den Einsatz der CTA zur Feststellung eines zerebralen Zirkulationsstillstandes gelten auch in der 5. Fortschreibung ab dem 18. Lebensjahr, da bis zum Abschluss der Überarbeitung der Richtlinien keine Studienergebnisse über den Einsatz der CTA zur Feststellung eines zerebralen Zirkulationsstillstandes bei einem größeren Kollektiv von Kindern vorlagen. Im Juni dieses Jahres wurde nun eine erste Studie bei 50 Kindern zu diesem Thema publiziert [11]. Grundsätzlich zeigte sich hierbei, dass die CTA auch bei Kindern zur Feststellung des zerebralen Zirkulationsstillstandes eingesetzt werden kann. Aufgrund der bei Kindern im Vergleich zu Erwachsenen anderen physiologischen Bedingungen empfehlen die Autoren jedoch die Bestätigung ihrer Ergebnisse durch weitere Studien. Für eine Aufnahme in die kommenden Richtlinien wäre dies sicher förderlich.

## Zusammenfassung

Die im Rahmen der 4. Fortschreibung eingeführten Kriterien zur Durchführung der CTA zum Nachweis des zerebralen Zirkulationsstillstandes und damit als ergänzendes Verfahren zur Feststellung des irreversiblen Hirnfunktionsausfalles haben sich bewährt. Mit Veröffentlichung der 5. Fortschreibung kann die CTA bei PatientInnen unter ECMO-Therapie nur – mit Präsenz eines erfahrenen ECMO-Spezialisten – bei der vv-ECMO eingesetzt werden. Für den Einsatz bei der va-ECMO ist dieses Verfahren nicht zugelassen. Bei PatientInnen mit einer Kalottenläsion kann die CTA – bei abhängig vom Ausmaß der Läsion reduzierter Sensitivität – zuverlässig eingesetzt werden. Hinsichtlich der Festlegung eines Grenzwertes für den Nachweis eines Stasis filling, eines potenziellen Zeitfensters für den Einsatz der CTA oder den Einsatz bei Kindern sind noch weitere Studien erforderlich.
